# *Monilinia* Species Causing Brown Rot of Peach in China

**DOI:** 10.1371/journal.pone.0024990

**Published:** 2011-09-27

**Authors:** Meng-Jun Hu, Kerik D. Cox, Guido Schnabel, Chao-Xi Luo

**Affiliations:** 1 Department of Plant Pathology, College of Plant Science and Technology and the Key Lab of Crop Disease Monitoring & Safety Control in Hubei Province, Huazhong Agricultural University, Wuhan, People's Republic of China; 2 Department of Plant Pathology and Plant–Microbe Biology, New York State Agricultural Experiment Station, Cornell University, Geneva, New York, United States of America; 3 Department of Entomology, Soils, and Plant Sciences, Clemson University, Clemson, South Carolina, United States of America; University of British Columbia, Canada

## Abstract

In this study, 145 peaches and nectarines displaying typical brown rot symptoms were collected from multiple provinces in China. A subsample of 26 single-spore isolates were characterized phylogenetically and morphologically to ascertain species. Phylogenetic analysis of internal transcribed spacer (ITS) regions 1 and 2, glyceraldehyde-3-phosphate dehydrogenase (*G3PDH*), β-tubulin (*TUB2*) revealed the presence of three distinct *Monilinia* species. These species included *Monilinia fructicola*, *Monilia mumecola*, and a previously undescribed species designated *Monilia yunnanensis* sp. nov. While *M. fructicola* is a well-documented pathogen of *Prunus persica* in China, *M. mumecola* had primarily only been isolated from mume fruit in Japan. Koch's postulates for *M. mumecola* and *M. yunnanensis* were fulfilled confirming pathogenicity of the two species on peach. Phylogenetic analysis of *ITS*, *G3PDH*, and *TUB2* sequences indicated that *M. yunnanensis* is most closely related to *M. fructigena*, a species widely prevalent in Europe. Interestingly, there were considerable differences in the exon/intron structure of the cytochrome b (*Cyt b*) gene between the two species. Morphological characteristics, including spore size, colony morphology, lesion growth rate, and sporulation, support the phylogenetic evidence suggesting the designation of *M. yunnanensis* as a new species. A new multiplex PCR method was developed to facilitate the detection of *M. yunnanensis* and differentiation of *Monilinia* spp. causing brown rot of peach in China.

## Introduction

China is the primarily producer of peaches (*Prunus persica* L. Batsch) worldwide providing approximately 43% of the world production. In addition, peaches and other agronomically important *Prunus spp.* are believed to have originated from Western China. Indeed, ancient records and archaeological findings, indicate that the domestication of *P. persica* may have occurred as early as 3000 BC [1]. Aside from *P. persica*, other *Prunus* spp. originating from Western China include *Prunus davidiana* (Carr.) Franch., *Prunus ferganensis* (Kost. and Rjab) Kov. and Kost., and *Prunus kansuensis* Rehd., reflecting great diversity of *Prunus* spp. in the region. In addition to serving as the origin of domesticated stone fruit, Western China is, likely to serve as the evolutionary origin of pathogens that cause diseases of *Prunus* spp.

The economically most important diseases of stone fruit are blossom blight and brown fruit rot caused by *Monilinia* spp. The earliest reports of brown rot on stone fruit in China were made in the 1920s [2]. At this time, fungal species were identified solely on the basis of morphology and as such were classified as *Monilinia fructigena* and *Monilinia laxa* [2], [3], [4], [5]. It was not until the 21^st^ century that the diversity of *Monilinia* species in China was more extensively characterized [6], [7]. However, many PCR-based diagnostic tools used to distinguish *Monilinia* spp. in Europe and the Americas, failed to differentiate the morphological species believed to be present in China [8] suggesting that other undescribed species may be present. To date, however, completely thorough investigations into the species causing brown rot of peach in China have not been undertaken. Please note that although only anamorph of several species are presented and discussed in the manuscript, we will use the teleomorph designation, *Monilinia*, when referring to the genus in order to be consistent with the nomenclature preference of recent literature on this organism. We will however, refer to the anamorph genus name *Monilia* when describing the new species.

To date, only three species of *Monilinia* have been found to occur on *Prunus* species worldwide; *Monilinia fructicola* (G. Winter) Honey, *Monilinia fructigena* (Aderhold & Ruhland) Honey, and *Monilinia laxa* (Aderhold & Ruhland) Honey. While *M. fructicola* is widespread in the Americas, and parts of Europe and Asia [9], *M. laxa* and *M. fructigena* are the primary species causing brown rot of peach in Europe [10]. However, all three *Monilinia* spp. have also, been reported in China [2], [3], [4], [5], [6], [11]. In addition to the three ubiquitous species, two additional species were reported recently in China. These include *M. polystroma*, which was documented to cause brown rot of *Malus* spp., *Pyrus* spp. and *Prunus* spp. (but not *Prunus persica*) [7], [12], and *M. mumecola*, which was initially isolated in Japan from mume (*Prunus mume*) in 1982 [13], [14], and later classified as a separate species [15]. *M. mumecola* was described as the causal agent of the brown rot of papaya in Hubei, China in 2009 [16].

Given the evolutionary history of *Prunus* sp, and the extensive history of *Monilinia* species reporting and reclassification in Asia, there is potential for both the identification of new species, and improved taxonomic delineation of existing species. Hence, an in-depth investigation of species prevalence using modern phylogenetic tools could be warranted to better understand disease concerns as they relate to species identity in the intensive modern production systems of peach and nectarine in China. The objectives of this study were to: 1) Survey and describe the *Monilinia* species attacking peach in the primary production regions of China; 2) morphologically and phylogenetically characterize a subset of isolates representing each species identified from each region; and 3) develop a rapid, PCR-based method to differentiate the previously documented, and newly described *Monilinia* species attacking peach in China.

## Materials and Methods

### Ethics statement

Hundreds of samples were collected in this study and sampling was always conducted with the approval of the owners of the fields.

### Collection of *Monilinia* isolates

Peach and nectarine (*Prunus persica* var. nectarine) fruit sporulating with *Monilinia* were collected from twenty-nine commercial orchards and one experimental orchard from nearly all of the major peach production provinces in China including Beijing (23 isolates), Shandong (12 isolates), Zhejiang (4 isolates), Fujian (11 isolates), Shanxi (4 isolates), Gansu (3 isolates), Hubei (36 isolates) and Yunnan (52 isolates). Single spore isolates were obtained as described previously with slight modifications [17]. Briefly, individual conidia dispersed on potato dextrose agar (PDA; 200 ml juice from 200 g potato, 20 g dextrose, and 18 g agar L^−1^) were excised with a thin glass needle under a COIC XSZ-4G compound light microscope (ChongQing Opitical & Electrical Instrument Co., Ltd, Chongqing, China) and transferred to clean PDA. After conidia germinated, individual colonies were transferred but only one single-spore isolate was maintained for each infected fruit sample. All of the 145 isolates collected were identified to species by morphological observation and PCR identification (data now shown). Twenty isolates were selected for phylogenetic and 11 for morphological analysis, respectively with an approximately equal number representing each species identified. The selected isolates represented all of the different in vitro phenotypes and geographical locations included in this study (Table S1). For long-term storage, isolates were stored at −20°C on filter paper (Haier, Shandong, China) until further use. Briefly, isolates were allowed to grow on filter paper discs (5 mm in diameter) placed on PDA and incubated at 22°C in darkness. After 4 days, discs with mycelium were removed and placed into a desiccator with silica gel for 7 days. Discs were transferred into 1.5 ml sterile centrifuge tubes with desiccated silica gel at the bottom, and stored at −20°C. For each experiment in the study, a new culture was started from a stored filter disc.

An additional 18 isolates of known species (Table S1) used as morphological and phylogenetic standards for *M. laxa* and Chinese *M. fructicola* were either on hand or provided by Dr. Imre J. Holb (Department of Plant Protection, University of Debrecen, Debrecen, Hungary) and Dr. Xili Liu (College of Agriculture and Biotechnology, China Agricultural University), respectively. In total, 37 isolates (Table S1) representing five species and three continents were used for phylogenetic analysis, and a further subset of this collection was selected for morphological and pathological characterization. An isolate of *Botryotinia fuckeliana* from a Hubei province peach tree, and an isolate of *Sclerotinia sclerotiorum* from a Hubei province canola (*Brassica napus* L.) plant were used to validate the genera specificity of diagnostic primer sets.

### DNA extractions

All representative isolates (Table S1) selected for phylogenetic analysis were grown on PDA at 22°C for 5 days in the dark. Single agar plugs containing actively growing mycelium were taken from the periphery of the advancing colonies and transferred to 250-ml flasks containing 40 ml of PDB (200 ml juice from 200 g potato and 20 g dextrose per liter). Flasks were shaken at 120 rpm for 4 days at 22°C. The mycelium was then removed from the broth, rinsed under sterile deionized water, and genomic DNA was subsequently extracted using the Easypure Plant Genomic DNA Extraction Kit (TransGen Biotech, Beijing, China) according to the manufacturer's instruction.

### Sequencing of the ITS regions and *G3PDH* and *TUB2* gene fragments

From representative isolates (Table S1), the ITS regions and fragments of the *G3PDH* and *TUB2* gene were sequenced for phylogenetic analysis. The ITS1-5.8S-ITS2 region was amplified from genomic DNA with primer pair ITS1/ITS4 [18]. Based on *G3PDH* gene sequences from *M. fructicola* (EF367148) and *M. fructigena* (AJ705043), a primer pair Mon-G3pdhF/Mon-G3pdhR was designed to amplify a 786 bp fragment (approximately 70%) of the *G3PDH* gene. Similarly, primer pair Mon-TubF1/Mon-TubR1 was designed based on the *TUB2* sequences from *M. fructicola* (AY283679) and *M. laxa* (AY349149) to amplify a 1630 bp fragment (approximately 92%) of the *TUB2* gene. All primers used in this study are listed in table 1. PCR amplification of both genes was performed for all isolates in 50 µl reaction volumes containing 1× PCR buffer (TransGen Biotech, Beijing, China), 20 ng template DNA, 0.4 µM of each primer, 200 µM of each dNTP, 2.5 unit of Easy Taq DNA polymerase (TransGen Biotech, Beijing, China). All amplifications were performed in an “iCycler” thermal cycler (Bio-Rad Laboratories Inc., Hercules, CA). The amplification parameters for the ITS, consisted of an initial denaturation at 94°C for 3 min followed by 30 cycles of 94°C for 1 min, 55°C for 1 min and 72°C for 2 min, and a final elongation step of 72°C for 5 min. The parameters for amplifying *G3PDH* and *TUB2* gene fragments were largely identical with ITS amplification, except for the annealing temperature, which was reduced to 50°C. PCR products were extracted for sequencing from agarose gels using a DNA Gel Extraction Kit (Axygen, Hangzhou, China) according to the manufacturer's instructions. Sequencing was conducted at Beijing Genomics Institute (BGI; Shenzhen, China).

**Table 1:** Primers used for PCR amplification and sequencing.

Primer Name	Primer Sequence (5′→3′)	Description
Mon-G3pdhF	ACGGTCAATTCAAGGGTGAT	To amplify the partial fragment of *G3PDH* in *Monilinia* spp
Mon-G3pdhR	ATCGAAGATGGAGGAGTGGT	To amplify and sequence the *G3PDH* fragment
Mon-TubF1	ATGCGTGAGATTGTACGTAT	To amplify and sequence the β-tubulin fragment in *Monilinia* spp
Mon-TubR1	GTACCAATGCAAGAAAGCCT	Same as Mon-TubF1
ITS1^a^	TCCGTAGGTGAACCTGCGG	To amplify ITS region
ITS4^a^	TCCTCCGCTTATTGATATGC	To amplify and sequence ITS region
PRC Laxa-F1	ATGAGAATTTTTAAAAGTCATCCC	Amplified and sequenced fragment 1
PRC Laxa-R1	CTAATGTTCTAGGTGCTCTG	Same as PRC Laxa-F1
PRC Laxa-F2	GCGTGATGTTAACAATGGATG	Amplified and sequenced fragment 2
Check R3	CAGGAACAGGCAGAATACA	Amplified fragment 2
KES 1238^b^	AGCTTTCCTGGGTTTGTCAAA	Amplified and sequenced fragment 3
KES 1261^b^	TCCAATTCATGGTAYAGCACTCATA	Amplified fragment 3
PRC Laxa-F3	GCAACTGTGATCACCAACCT	Amplified and sequenced fragment 4
P450intron6-2-rev^c^	AGTTCAACTCAGATCTAAAGATACCTC	Same as PRC Laxa-F3
P450intron6-2-fwd^c^	AGGTGAGTAGGAAATACAGATAAATG	Amplified and sequenced fragment 5
PRC Laxa-R4	TTATCTACTAGGCTTTTC	Same as P450intron6-2-fwd
PRCmon-F	ATCTCCAACGCTTCTTGCAC	Specific primer for *Monilinia* spp. from *G3PDH*
PRCmon-R	CTTCTTGACGACAGCCTTGA	Same as PRCmon-F
*Cola*-F	CTGTATGATGACCGAGAAGG	Species-specific primer for *M. fructicola*
*Ensis*-F	GGAAACCAAGTGGTTGAGAT	Species-specific primer for *M. yunnanensis*
*Mume*-F	AAAGGTAGAAGACATCTTAAGG	Species-specific primer for *M. mumecola*
Mon-R	ATCTCCAAGATCCGTGAGGAG	Common reverse primer for *M. fructicola*, *M. yunnanensis*, and *M. mumecola* from β-tubulin gene

a White et al 1990.

b Miessner and Stammler 2010.

c Hily et al 2010.

### Construction of phylogenetic trees

Phylogenetic analysis on the representative isolates (Table S1) was performed for the noncoding renal region (ITS1-5.8S-ITS2) and a combined data matrix of the two coding loci (*G3PDH* and *TUB2*) respectively. Multiple alignments were conducted using DNASTAR (DNASTAR Inc., Nevada City CA) and CLUSTAL X 1.81 [19], [20]. For constructing the ITS phylogenetic tree, sequences of *B. fuckeliana* (FJ791158) was used as out-group reference species. Strain SAS56 of *B. fuckeliana* (GenBank accessions: AJ705006 and Z69263) was used as the outgroup species in the construction of the *G3PDH* and *TUB2* phylogenetic trees. Maximum parsimony (MP) method and neighbor-joining (NJ) method were used to carry out phylogenetic constructions using MEGA version 4.0 [21]. For the MP tree, the following settings were used: heuristic search using close neighbor interchange (CNI; level = 1), and branch swapping method with initial trees generated by random addition (10 reps). A maximum composite likelihood model was used to generate the NJ tree. A complete deletion option was used to treat gaps/missing data and the reliability of clusters was evaluated by bootstrapping with 1000 replicates.

### Cloning and analysis of cytochrome b sequences from *M. yunnanensis* and *M. mumecola*

To provide further evidence that *M. yunnanensis* and *M. mumecola* were phylogenetically distinct from known *Monilinia* species of peach, the mitochondrial cytochrome b (C*yt b*) genes were cloned and sequences were compared to those of known species. Genomic DNA was extracted from 1-week-old mycelial cultures of *M. yunnanensis* (isolate YQG10-6c) and *M. mumecola* (isolate ML-1a) using Trizol reagent (Invitrogen, Carlsbad, CA) according to the manufacturer's recommendations. Total RNA was isolated from 5-day-old mycelial cultures of *M. yunnanensis* (isolates SBG10-3a, YKG10-61c, KY-1, QJ-2a and YQG10-6c) and *M. mumecola* (ML-1a and HXL10-1a) using the RNeasy Plant Mini Kit (Qiagen, Valencia, CA) according to the manufacturer's instructions. RNA integrity was verified by resolving on a 1% agarose gel. Quantification of all nucleic acids was carried out using a NanoDrop 1000 spectrophotometer (Thermo Fisher Scientific Inc., Waltham, MA). First-strand cDNA synthesis was conducted using 500 ng total RNA from each of the *M. yunnanensis* and *M. mumecola* isolates with an oligo-dT primer and Superscript III reverse transcriptase (Invitrogen) in a final volume of 10 µl according to the manufacturer's recommendations. Subsequent amplifications of *Cyt b*-specific cDNAs were performed using 1 µL cDNA and the Platinum PCR Supermix High Fidelity system (Invitrogen) in a final volume of 25 µL. Primers MoniliniaP450ATG-fwd (ATG AGA ATT TTT AAA AGT CAT CCC T) and MoniliniaP450STOP-rev (TTA TCT ACT AGG CTT TTC TTT AGT TAA TAC) were designed to amplify the *Cyt b* sequence from translational start codon to translational stop codon based on the previously published *Cyt b* sequence from the closely related *B. fuckeliana* (GenBank accession AB262970). In each case, reactions were incubated at 94°C for 2 min, followed by 32 cycles of 94°C for 30 s, 55°C for 30 s and 68°C for 1.5 min, with a final elongation step of 68°C for 5 min.

The full-length *Cyt b* gene (from translational start codon to translational stop codon) was amplified from genomic DNA of *M. yunnanensis* (YQG10-6c) in a single PCR reaction using primers MoniliniaP450ATG-fwd and MoniliniaP450STOP-rev. PCR amplifications were carried out with approximately 200 ng template DNA using the Platinum PCR Supermix High Fidelity system. Thermal parameters utilized were identical to those described for the cloning of *Cyt b* cDNA, except for the elongation time, which was increased to 13 min, and the number of cycles, which was increased to 35.

In the case of *M. mumecola* (isolate ML-1a), five successive PCR reactions were performed to achieve the full-length *Cyt b* gene sequence. The five primer pairs for the reactions are listed in Table 1. Thermal parameters for amplifying each fragment were as follows with 35 cycles each: 20 sec at 95°C, 30 sec at 52°C, and 5 min at 68°C (Fragments 1 and 4); 40 sec at 94°C, 50 sec at 50°C, and 3 min at 72°C (Fragments 2 and 5); 15 sec at 95°C, 30 sec at 55°C, and 5 min at 68°C (Fragment 3).

All PCR products were gel-purified using the Wizard SV Gel and PCR Clean-Up system (Promega, Madison, WI) and were cloned into the pGEM-T easy vector (Promega). All sequencing was carried out using Big Dye Terminator chemistry and AmpliTaq-FS DNA Polymerase (Applied Biosytems, Foster City, CA) using the Applied Biosystems Automated 3730xl DNA Analyzer at the Cornell University DNA Sequencing facility in Ithaca, NY. In the case of the *Cyt b* gene sequence, a primer walking strategy was utilized for sequencing due to its large size (a complete listing of the primer sequences are available upon request). All nucleotide sequences were aligned manually using Clustal W [22] and BioEdit version 7.0.8.0 [23], and were compared to previously reported sequences using BLAST [24].

### Colony morphology, mycelial growth rate, sporulation, and conidial morphology of *Monilinia* species

At least four representative isolates from each species (*M. yunnanensis*, isolates SBG10-3a, YQG10-6c, SM09-1a, KY-1; *M. fructigena*, isolates SL10, Mfg2-GE-A, Mfg4-GY-A, Mfg5-SP-A; *M. mumecola*, isolates ML-1a, ML-1c, HWL10-13b, HXL10-1a; *M. laxa*, isolates EBR Ba11b, GEARI 3c2a, BEK-SZ, BSZGY-SZ-1 and *M. fructicola*, isolates ZM09-2a, MSA9, 0907-a, SC.Dap3, GA.Bmpc5, SC.Egpc8) were selected for in-depth morphological and physiological characterization. To obtain uniform colonies for each isolate, 6 mm-diameter plugs with actively growing mycelium were removed from the periphery of a 4-day-old colony grown on PDA and placed in the center of plastic Petri dishes (90 mm diameter) containing PDA and incubated at 22°C in the dark. Mycelial growth rates were determined on both PDA and V8 (200 ml V8 juice and 20 g agar/l) media. Growth rates of isolates were determined by incubating isolates at 22°C in darkness and measuring colony diameters (excluding the transfer plug of 6 mm) every 2 days until hyphae were within 2 mm of the edge of the 90 mm in diameter Petri dish. Growth rates were expressed as mm of growth per day and the mean of 3 replicate colonies were used to represent each isolate. Sporulation was quantified on PDA, V8, and peach fruit. Isolates were incubated on the two media at 22°C in darkness for 9 days before conidia were rinsed off in 2 ml of sterile water for spore counts. Inoculation of peach fruit for determination of sporulation was identical to the method for determining pathogencity on fruit (see below). After 9 days of incubation, 2 ml of sterile water was added to the surface of sporulating colonies and conidia were gently dislodged using sterile plastic inoculation loops. The concentration of conidia in the suspensions from media and fruit was determined using a haemocytometer, and mean counts of three replicate samples were determined for each isolate and medium (including fruit) combination. In addition of the quantification of conidia, the conidial size, and germ tube morphology were also determined for each of the representative isolates. Conidia were harvested from sporulating peach fruit and spread onto shallow PDA media (<3 mm thick for maximal optical density) using sterile cotton swabs. PDA media containing conidia was incubated at 22°C in the dark for 5 hrs, and subsequently, the length and width of conidia and germ tubes were measured with a stage micrometer using a COIC XSZ-4G compound light microscope (ChongQing Opitical & Electrical Instrument Co.) at 400× magnification. For each isolate a minimum of 100 conidia and germ tubes were measured. Least significant difference (LSD) tests were conducted using DPS Data processing system 3.01 [25].

### Pathogenicity of *Monilina* species

The pathogenicity of representative isolates of *M. yunnanensis*, *M. fructigena*, *M. mumecola*, *M. laxa* and *M. fructicola* on peach was determined. Commercially mature peach fruit (cv. ‘Zhonghua 2’) of similar size were collected, surface-sterilized with 75% ethanol and rinsed with sterile water to remove any pesticide residues prior to inoculation. A 5 mm plug taken from the periphery of a 4-day-old colony grown on PDA was inserted into a 5 mm deep hole created in the periderm of the fruit using a 5 mm cork borer. Fruit were subsequently transferred into non-branded plastic plant propagation trays (l×w×h = 50×30×20 cm) and covered with a transparent plastic lid. Wet paper towels were placed on the bottom of the trays to maintain near 100% humidity. Trays were incubated at 22°C, 97% RH (relative humidity) under a 14 hr light/10 hr dark regime. Lesion diameters were measured after 2 and 4 days of incubation. Afterwards, the lid was removed and RH was adjusted to 75% to promote dehiscence of conidial chains. One day later, conidia were collected with a sterile swab and transferred into 15–20 ml distilled water. The suspension was filtered through a double layer of gauze, and the number of conidia was determined using a haemocytometer and a COIC XSZ-4G compound light microscope. Lesion development and sporulation were determined on six replicate fruit per isolate, and the entire pathogenicity assay was repeated. Statistical analyses were performed by DPS Data processing system 3.01 [25].

### Evaluation of PCR-based methods to distinguish *Monilinia* species from China

Six PCR based methods previously developed to distinguish *Monilinia* spp. were evaluated for applicability to differentiate the Chinese *Monilinia* species pathogenic on peach. Primer sets included ITS1Mfcl/ITS4Mfcl, ITS1Mlx/ITS4Mlx, ITS1Mfgn/ITS4Mfgn [26]; IMfF/IMfR, MLF2/MLR2 [27], [28]; MO368-5, MO368-8R, MO368-10R, and Laxa-R2 [29]; *IColaS*/*IColaAS*, *IGenaS*/*IGenaAS*, and *ILaxaS/ILaxaAS* [30]; KES 1238/KES 1261 [31], as well as P450intron6-2-fwd/P450intron6-2-rev [32]. All PCR procedures were performed as described in the associated references.

### Development of a PCR-based method to distinguish Chinese *Monilinia* species

Based on the aligned sequences of *G3PDH* from Chinese *Monilinia* isolates, *B. fuckeliana* (AM231159), and *S. sclerotiorum* (AJ705044), primers PRCmon-F and PRCmon-R were designed to differentiate the *Monilinia* species causing brown rot of peach. Primers based on *TUB2* sequences were designed to distinguish Chinese *Monilinia* species from each other. Reverse primer Mon-R and species-specific forward primers, *Cola*-F, *Ensis*-F and *Mume*-F were designed to differentiate *M. fructicola*, *M. yunnanensis* and *M. mumecola*, respectively. Closely related *B. fuckeliana* and *S. sclerotiorum* were included in all PCR experiments as out-group negative controls [33]. PCR reactions were carried out in a volume of 25 µl containing 1× PCR buffer (TransGen Biotech, Beijing, China), 200 µM of each dNTP, 20 ng template DNA, 0.2 µM of each primer, and 1 unit of Easy Taq DNA polymerase (TransGen Biotech, Beijing, China). The PCR amplification program consisted of an initial denaturation at 94°C for 3 min followed by 35 cycles of 30 sec at 94°C, 30 sec at 58°C, and 40 sec at 72°C and a final extension step at 72°C for 5 min. Products were resolved on 1.2% agarose gel (AGAROSE G-10, GENE COMPANY, Hong Kong, China) in 0.5×TBE buffer for 1 h at 100 v. Gels were stained with ethidium bromide, and visualized using an Alphalmager® EP image acquisition system (Alpha Innotech, Santa Clara, CA, USA).

## Results

Based on nucleotide sequence comparisons of the noncoding ribosomal ITS regions and the coding regions of *G3PDH* and *TUB2* genes, and unique morphological features (see below), several Chinese isolates were found to be sufficiently distinct from other known *Monilinia* species as to potentially warrant classification of a new species, which we designated *Monilia yunnanensis*. Detailed description of the proposed species and summary to distinctive features is provided below. The remainder of the isolates was classified as either *M. fructicola* with highest similarity to those from the United States or *M. mumecola* with highest similarity to those from China.

### Analysis of internal transcribed spacer (ITS) regions 1 and 2

**Figure 1:**
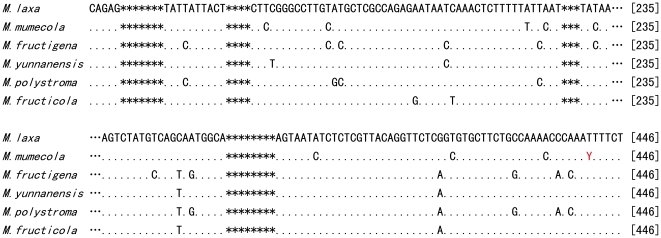
Sequence alignment of the ITS1 and ITS2 regions of ribosomal DNA (rDNA) of *Monilinia* spp. The sequence of *M. polystroma* (accession no. Y17876) was obtained from Genbank. Each Symbol ‘*’ represents conserved regions of 5 bp in length. while ‘…’ represent conserved regions of 95 bp in length. “Y” in bold indicates both cytosine(C) and thymine (T) were present in this position in *M. mumecola*.

The ITS1-5.8S-ITS2 sequences of 7 isolates (Table S1) were identical with *M. mumecola* (Genbank accession nos. AB125620, AB125613 and AB125614). Within each species, the ITS sequences of *M. yunnanensis*, *M. fructigena*, *M. fructicola*, and *M. laxa* isolates were 100% identical to one another. Interestingly, all of the *M. mumecola* isolates collected from peach had a cytosine (C) at the 442 position while all isolates from nectarine displayed a thymine (T) at this location (Fig. 1). Between the four species, there were considerable nucleotide variations. A total of eleven base pair differences were observed between *M. yunnanensis* and the next closest species *M. fructigena*. A minimum of eight base pair differences were observed between *M. mumecola* and the next closest species *M. laxa* from Europe and the United States. All *M. fructicola* isolates regardless of the origin had identical ITS sequences. There were 18 or 19 (depending on the origin of the isolate) variations between *M. mumecola* and *M. polystroma*, and 10 variations between *M. yunnanensis* and *M. polystroma*.

**Figure 2:**
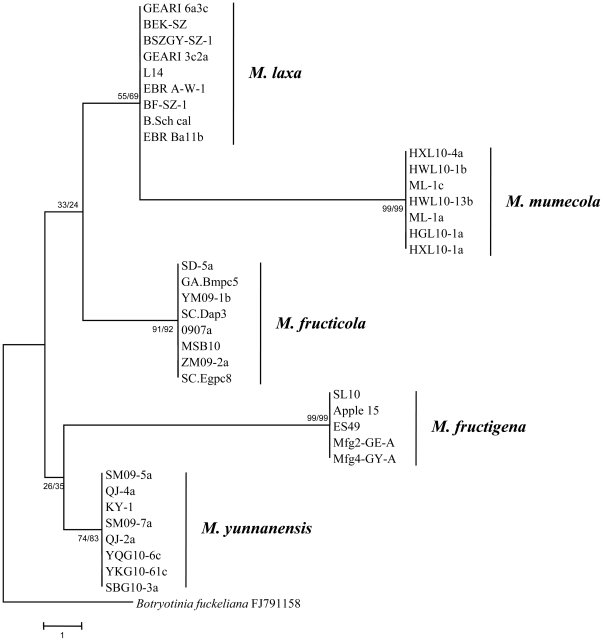
Phylogeny of the rDNA region ITS1-5.8S-ITS2. Shown is the most parsimonious tree for 37 *Monilinia* isolates and one outgroup species (*Botryotinia fuckeliana*), with 437 characters, out of which 18 were parsimony informative. The numbers labeled at each node indicate the bootstrap (BS) percentages (N = 1000) supporting individual branches: BS value from the MP test/BS value from the neighbor-joining (NJ) test.

Phylogenetic analysis was conducted with *Monilinia* species causing brown rot of peach and nectarine in China (thus *M. polystroma* was excluded). The ITS data set consisted of 38 fungal isolates including the outgroup fungus *B. fuckeliana*. There were a total of 437 nucleotide positions included in the final dataset, 18 of which were parsimony informative. MP (Fig. 2) and NJ (data not shown) analyses resulted in similar topologies. Both phylogenetic trees revealed distinct clusters for each species. From the topology of the ITS tree, *M. yunnanensis* isolates were most closely related to *M. fructigena*, whereas *M. mumecola* isolates were most closely related to *M. laxa*. The ITS sequences for *M. fructicola* isolate 0907-a, *M. laxa* isolate BF-SZ-1 obtained from Europe , *M. fructigena* isolate SL10 from Europe, and *M. yunnanensis* isolate KY-1 were deposited in GenBank under accession numbers HQ908789, HQ908790, HQ908791, and HQ908788 respectively. The sequences of *M. mumecola* isolates ML-1a from peach and HXL10-1a from nectarine were submitted under accession numbers HQ908786 and HQ908787, respectively. Unfortunately, the phylogenetic tree based on ITS sequences revealed many low bootstrap values, and as such, provided a weak indication of genetic relationships between some of the clades (Fig. 2).

### Construction of phylogenetic trees using *G3PDH* and *TUB2* sequences

**Figure 3:**
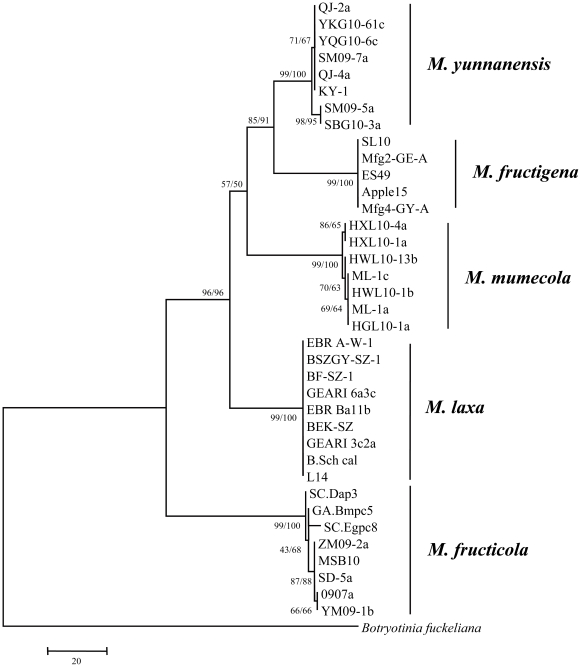
Phylogeny of 38 isolates of *Monilinia* spp. and *Botryotinia fuckeliana*. Maximum parsimony (MP) tree inferred from the data set containing the combined DNA sequences of *G3PDH* and *TUB2*, with 2215 characters, 172 of which were informative. The numbers labeled at each node indicate the bootstrap (BS) percentage (*N* = 1000): BS value from the MP test/BS value from the neighbor-joining (NJ) test.

Phylogenetic trees were generated from a data set of combined partial sequences of the *G3PDH* and *TUB2* genes from the same above-mentioned 38 isolates including the outgroup species *B. fuckeliana*. There were a total of 2215 nucleotide positions included in the final dataset, 172 of which were parsimony informative. MP and NJ analysis of the combined DNA sequences generated two distinct phylogenetic trees with a similar topological structure. Hence, the MP phylogeny for the combined partial *G3PDH* and *TUB2* sequences (Fig. 3) was used for drawing conclusions regarding species. In both MP and NJ (data not shown) phylogenetic trees, isolates of the three European and North American *Monilinia* species (*M. fructicola*, *M. fructigena* and *M. laxa*), and the two Chinese species *M. yunnanensis* and *M. mumecola* formed monophyletic clades within a larger derived clade within the *Monilinia* genus. *M. yunnanensis* isolates were more closely related to European *M. fructigena* isolates than to *M. mumecola* isolates. *M. fructicola* was the most basal clade, while the clades of the other species were more derived but shared a single common ancestor with *M. fructicola*. *G3PDH* sequences were deposited in GenBank under accession nos. HQ908777, HQ908778 & HQ908779 for *M. fructicola*, HQ908781 for *M. laxa* from Europe, HQ908784 & HQ908785 for *M. mumecola*, HQ908780 for European *M. fructigena*, and HQ908782 & HQ908783 for *M. yunnanensis*. *TUB2* sequences were deposited under accession nos. HQ908768, HQ908769 & HQ908770 for *M. fructicola*, HQ908772 for *M. laxa* from Europe, HQ908774, HQ908775 & HQ908776 for *M. mumecola*, HQ908771 for European *M. fructigena*, and HQ908773 for *M. yunnanensis*.

### Comparison of the *Cyt b* sequences from *Monilinia* species

**Figure 4:**
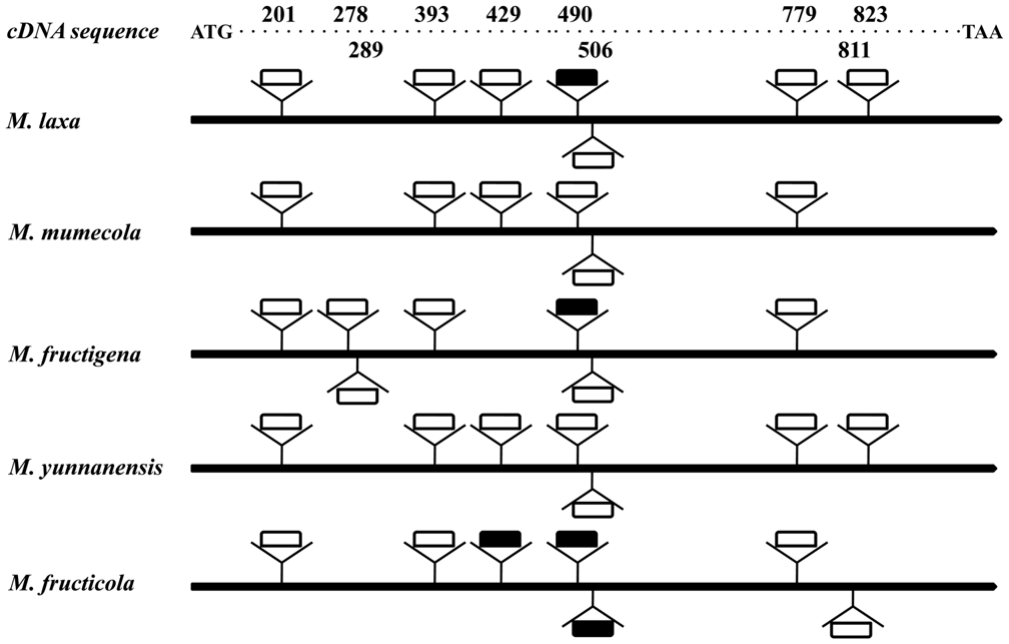
Exon/intron organization of the *Cyt b* gene from five *Monilinia* species. Schematic of *Cyt b* gene exon and intron organizations from *Monilinia* species showing intron positions and intron sequence similarities. Identical intron symbols at the same nucleotide position indicate high sequence similarity. For example, at position 490, *M. laxa* and *M. fructigena* had homologous introns, and *M. mumecola*, *M. yunnanensis* and *M. fructicola* had homologous introns but the two groups of introns did not share high sequence similarity.

*Cyt b* sequences were shown phylogenetically informative among *Monilinia* spp. in our previous study [32]. Therefore, *Cyt b* genes were isolated from *M. yunnanensis* and *M. mumecola* and compared to *M. fructicola*, *M. laxa* and *M. fructigena*. The *Cyt b* gene from *M. yunnanensis* was 12632 bp in length with 7 introns separating the exons; the same gene from *M. mumecola* was 14203 bp in length with 6 introns separating exons (Genbank accession nos. HQ908793 and JN204425, respectively; Fig. 4). The *Cyt b* coding sequences were both 1176 bp in length and shared 99% identity.

The *Cyt b* gene coding sequences were identical for all five *M. yunnanensis* isolates (GenBank accession no. of isolate YQG10-6c is HQ908792) and shared 99% identity with *M. fructigena* from Europe (Genbank accession no. GU952818), 99% identity with *M. laxa* from the United States (GU952816), and 98% identity with *M. fructicola* from the United States (GU952814). The *Cyt b* gene coding regions were identical for the two *M. mumecola* isolates sequenced (GenBank accession no. for isolate ML-1a is JN572107) and shared 99% identity with *M. laxa* from the United States (GU952816), 99% identity with *M. fructigena* from Europe (GU952818), and 98% identity with *M. fructicola* from the United States (GU952814). The *Cyt b* gene structure of *M. laxa*, *M. mumecola*, *M. fructigena*, *M. yunnanensis* and *M. fructicola* isolates differed in exon/intron organization (Fig. 4) in that the introns showed a patchy distribution. For example, in *M. yunnanensis*, the positions of five of the seven introns were identical to those of other *Monilinia* species [32]. Intron 1 of *M. yunnanensis* and *M. fructigena* was located at position 201, and introns 2, 5, and 6 of *M. yunnanensis* corresponded to introns 4, 6, and 7 of *M. fructigena* located at positions 393, 506 and 779, respectively (Fig. 4). Despite the fact that these homologous introns have high levels of identity (>98%) with one another, the sizes of these introns clearly differed between the two species. Interestingly, intron 4 of *M. yunnanensis* and intron 5 of *M. fructigena* were located at position 490 but the sequences did not share noticeable sequence similarity (Fig. 4). Moreover, introns 3 and 7 of *M. yunnanensis* and introns 2 and 3 of *M. fructigena* were located at different positions in the coding sequence and shared no noticeable sequence similarity (Fig. 4).

### Colony morphology, mycelial growth rate, sporulation, and conidial morphology of *Monilinia* species

**Figure 5:**
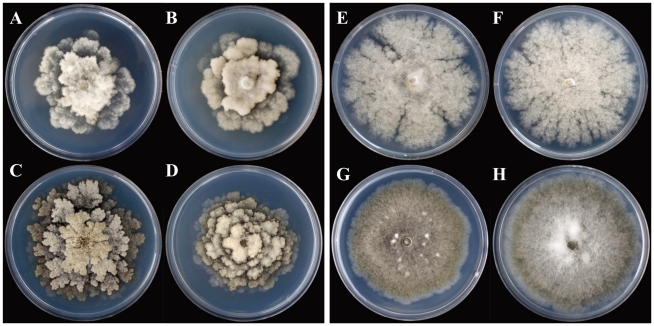
Characteristics of single spore isolates of *Monilinia* spp. grown on potato dextrose agar (PDA) at 22°C in darkness for 9 days. A–B colonies of *M. laxa* (isolates GEARI 3c2a and BSZGY-SZ-1). C–D colonies of *M. mumecola* (isolates HGL10-1a and ML-1a). E–F colonies of *M. fructigena* (isolates Mfg2-GE-A and Mfg4-GY-A). G–H colonies of *M. yunnanensis* (isolates QJ-4a and SM09-5a).

Colony morphology of *M. mumecola* was similar to *M. laxa* (Fig. 5A–D). These two species have a growth pattern distinct from any other species characterized by concentric rings of mycelium with lobbed margins. Despite a similar appearance, the colony color of *M. mumecola* tended to be gray-green compared to the gray *M. laxa* colony. Similarly, the colony color of *M. yunnanensis* isolates was gray-green compared to the gray *M. fructigena* colony (Fig. 5E–H). Fragmented radial colonies, which were frequently observed in *M. fructigena* isolates, were rarely observed in *M. yunnanensis* (Fig. 5E–H). After more than 10 days of incubation, most *M. yunnanensis* isolates started to develop stromata. However, stromatization was never observed in any of the *M. fructigena* isolates.

The mean and range of colony growth rates of *Monilinia* species on PDA medium were significantly different (P<0.05) between *M. fructicola* and *M. laxa*, *M. fructicola* and *M. mumecola* and between *M. fructigena* and *M. laxa*. *M. fructicola* isolates displayed the highest growth rates while *M. laxa* exhibited the lowest. On V8 medium, *M. mumecola* grew significantly (*P*<0.05) faster than *M. laxa* isolates. However, no significant differences (*P*>0.05) in growth rate were observed between *M. yunnanensis* and *M. fructigena*. In general, colony growth rates of all test isolates were found to be higher on V8 medium compared to PDA. On PDA medium, there were no significant differences in the number of conidia produced among *Monilinia* species (Table 2). On V8 medium only *M. fructicola* consistently sporulated, and the other species groups rarely produced conidia (Table 2).

**Table 2:** Colony growth rate and sporulation of *Monilinia* isolates on PDA and V8 media^z^.

Species	Colony growth rate (mm d^−1^)				Sporulation^x^			
	On PDA		On V8		On PDA		On V8	
	Mean^y^	Range	Mean	Range	Mean	Range	Mean	Range
*M. fructicola*	10.8±0.79a	8.3–12.7	12.0±0.24a	10.9–12.5	3.9±0.45a	2.8–5.3	5.2±0.14	4.6–5.4
*M. yunnanensis*	8.5±0.59abc	6.5–9.9	10.9±0.06ab	10.8–11	nd	-	nd	-
*M. fructigena*	8.9±1.02ab	6.8–10.8	10.2±0.82ab	7.8–11.5	nd	-	nd	-
*M. mumecola*	6.9±1.48bc	4.0–8.7	11.6±1.31a	9.7–14.1	2.5±0.85a	0–2.9	nd	-
*M. laxa*	6.0±0.58c	4.5–7.3	8.2±0.94b	5.4–9.5	1.8±0.91a	0–3.7	nd	-

z Average of at least 4 isolates from each species.

x log-transformed number of conidia per cm^2^; nd = not detected.

y Mean ± S.E.M (standard error of mean); values within the same column followed by the same letters are not significantly different based on the analysis of least significant difference (LSD) test at *P* = 0.05.

Differences in conidia size were apparent among species (Table 3). The conidia of *M. mumecola* were the largest on average, and those of the *M. laxa* were the smallest on average. The conidia of *M. yunnanensis* isolates were smaller on average compared with those of *M. fructigena* (Table 3). Consistent with that, the range of conidia sizes was unique for each of the new species and strikingly distinct from the genetically closest *Monilinia* relative. For example, the range of conidia size for *M. yunnanensis* was 10–21×7–12, whereas the range for *M. fructigena* was 12–31×7–17 (Table 3).

**Table 3:** Lesion growth rate, sporulation, and conidia size of selected *Monilinia* isolates on peach fruit.

Species	Lesion growth rate (mm d^−1^)		Num of conidia, cm^−2^		Conidia size, µm	
	Exp 1	Exp 2	Exp 1	Exp 2	Mean (L×W)	Range
*M. fructicola*	21.2±1.16c^z^	20.7±0.77b	6.3±0.11a	6.5±0.08a	16×10	10–19×7–14
*M. yunnanensis*	25.5±0.52ab	27.7±0.79a	2.6±0.34d	2.9±0.32d	15×9	10–21×7–12
*M. fructigena*	21.3±0.89c	23.6±1.04b	4.3±0.02b	4.2±0.07b	22×12	12–31×7–17
*M. mumecola*	22.4±0.79bc	23.5±1.30b	3.3±0.29cd	3.4±0.04cd	21×15	14–31×11–17
*M. laxa*	25.8±1.07a	27.3±0.21a	4.0±0.16bc	3.9±0.09bc	13×9	10–17×7–11

z Mean ± S.E.M (standard error of mean); values within the same column followed by the same letters are not significantly different based on the analysis of least significant difference (LSD) test at *P* = 0.05. Values were log transformed prior to statistical analysis.

**Figure 6:**
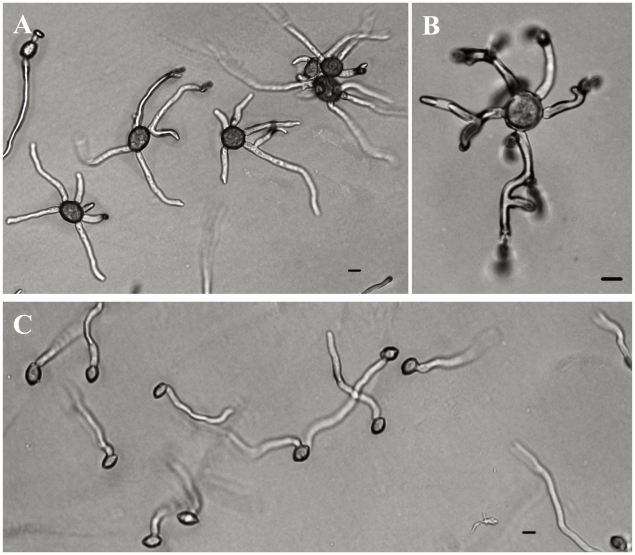
Germ tube morphology of *M. mumecola* (A–B) and *M. laxa* (C). Conidia were incubated on PDA at 22°C in the dark for 6 hours. Bar = 10 µm.

*M. mumecola* isolates often produced more than two germ tubes per conidium, which also appeared somewhat misshapen (Fig. 6A, B). By comparison *M. yunnanensis* and *M. fructigena* produced one or two germ tubes per conidium, and all *M. laxa* and *M. fructicola* isolates consistently produced one germ tube per conidium (Fig. 6C).

### Pathogenicity of *Monilina* species

**Figure 7:**
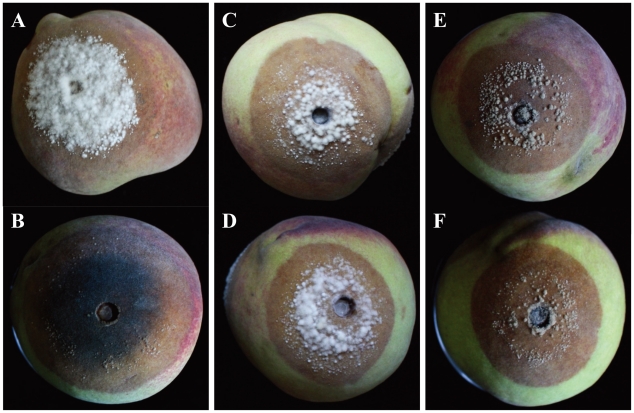
Symptoms of infection by *Monilinia spp.* on peach fruit (cv. ‘Zhonghua 2’) after 3 days of incubation at 22°C in 14 h light/10 dark regime. A–F, lesion resulting from infection by *M. mumecola*, *M. laxa*, *M. yunnanensis*, *M. fructigena*, Chinese *M. fructicola* and American *M. fructicola*, respectively.

All species tested (*M. yunnanensis*. *M. fructigena*, *M. mumecola*, *M. laxa* and *M. fructicola*) were pathogenic and sporulated on peach fruit. Koch's postulates were fulfilled by reisolating the fungus from symptomatic fruit and re-identifying the pathogen to the species level (data not shown). In each experimental run, significant differences (*P*<0.05) in average lesion growth rates were observed between *M. yunnanensis* and *M. fructigena* and between *M. mumecola* and *M. laxa* (Table 3). Similarly, the average number of conidia produced on lesions by *M. yunnanensis* and *M. fructigena* were significantly different (*P*<0.05), but not for *M. mumecola* and *M. laxa* (Table 3). Also, lesion morphology on peach fruit differed between *M. mumecola* and *M. laxa* (Fig. 7A, B). The lesions on fruit inoculated with *M. mumecola* developed a whiter, denser mycelium than those of produced by isolates of *M. laxa*, which produced sparse aerial mycelium with a black distinct lesion margin. *M. yunnanensis* and *M. fructigena* produced indistinguishable symptoms (Fig. 7C, D), which was also the case for *M. fructicola* isolates from China and the US (Fig. 7E, F).

### Evaluation of PCR-based methods to distinguish *Monilinia* species from China

PCR results of six methods designed to differentiate *Monilinia* spp. are shown in Table 4. None of the six molecular tools alone was able to distinguish all five species (*M. fructicola*, *M. fructigena*, *M. laxa*, *M. yunnanensis* and *M. mumecola*) from one another. *M. fructicola*, *M. fructigena*, and *M. laxa* isolates were reliably differentiated by the methods of Ioos et al. [26], Miessner and Stammler, [31], and Hily et al. [32]. However, neither of these methods was able to distinguish *M. yunnanensis* from *M. fructigena*. Likewise, the methods developed by Ioos et al. [26] and Ma et al. [27], [28] did not distinguish between *M. mumecola* and *M. laxa* and the method developed by Hily [32] did not distinguish *M. mumecola* from *M. fructicola*. Additionally, the methods developed by Miessner and Stammler [31], and Hily et al. [32] did not distinguish between *M. yunnanensis* and *M. laxa* (Table 4).

**Table 4:** PCR results of different diagnostic methods to distinguish *Monilinia* species^z^.

Isolates		Ioos et al., 2000			Ma et al., 2003, 2007		Cote et al., 2004			Gell et al., 2007			Miessner and Stammler, 2010			Hily et al., 2010		
Name	Taxon	A	B	C	A	C	A	B	C	A	B	C	A	B	C	A	B	C
0907a	*M. fructicola*	**+**	−	−	**+**	−	**+**	**−**	**−**	**+**	−	−	**+**	−	−	**+**	−	−
SD−5a	*M. fructicola*	**+**	−	−	**+**	−	**−**	**−**	**−**	**+**	−	−	**+**	−	−	**+**	−	−
ZM09−2a	*M. fructicola*	**+**	−	−	**+**	−	**+**	−	**−**	**+**	−	−	**+**	−	−	**+**	−	−
SC.Dap3	*M. fructicola*	**+**	−	−	**+**	−	−	−	−	**+**	−	−	**+**	−	−	**+**	−	−
SC.Egpc8	*M. fructicola*	**+**	−	−	**+**	−	−	−	−	**+**	−	−	**+**	−	−	**+**	−	−
QJ−2a	*M. yunnanensis*	−	**+**	**+**	−	**+**	−	−	−	−	−	−	−	−	**+**	−	−	**+**
SM09−7a	*M. yunnanensis*	−	**+**	**+**	−	**+**	−	−	−	−	−	−	−	−	**+**	−	−	**+**
SBG10−3a	*M. yunnanensis*	−	**+**	**+**	−	**+**	−	−	−	−	−	−	−	−	**−**	−	−	**+**
SL10	*M. fructigena*	−	**+**	**−**	−	**+**	−	**+**	−	−	−	−	−	**+**	−	−	**+**	−
Mfg2−GE−A	*M. fructigena*	−	**+**	−	−	**+**	−	**+**	−	−	−	−	−	**+**	−	−	**+**	−
ML−1c	*M. mumecola*	−	−	**+**	**+**	**+**	−	−	−	−	−	−	−	−	−	**+**	−	−
HGL10−1a	*M. mumecola*	−	−	**+**	−	**+**	−	−	−	−	−	−	−	−	−	**+**	−	−
HXL10−4a	*M. mumecola*	−	−	**+**	−	**+**	−	−	−	−	−	−	−	−	−	**+**	−	−
BSZGY−SZ−1	*M. laxa*	−	−	**+**	−	**+**	−	−	**+**	−	−	**+**	−	−	**+**	−	−	**+**
EBR Ba−1−1b	*M. laxa*	−	−	**+**	−	**+**	−	−	**−**	−	−	**+**	−	−	**+**	−	−	**+**

z A: *M. fructicola*–specific product; B: *M. fructigena*–specific product; C: *M. laxa*–specific product; ‘**+**’ and ‘−’ indicate the presence and absence of specific band patterns, respectively.

### Development of a molecular tool to distinguish Chinese *Monilinia* species affecting peach

**Figure 8:**
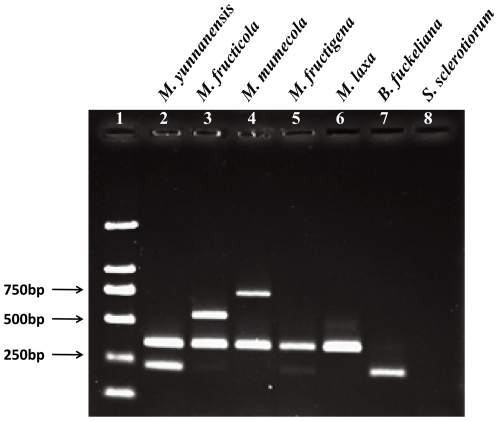
Multiplex polymerase chain reaction (PCR) results for five *Monilinia* spp. Trans 2K (TransGen Biotech, Beijing, China) molecular marker is shown in the first lane. Primer pair PRCmon-F/PRCmon-R were used to produce a 354 bp fragment (lanes 2–6) from all five *Monilinia* species, but not from other closely related genera (*Botrytinia*,lane 7; *Sclerotinia*, lane 8). Reverse primer Mon-R and species-specific forward primers *Ensis*-F, *Cola*-F, and *Mume*-F, produced amplicons 237 bp (lane 2), 534 bp (lane 3), or 712 bp (lane 4) in length from *M. yunnanensis*, *M. fructicola*, or *M. mumecola*, respectively.

A multiplex PCR method was developed to differentiate Chinese *Monilinia* species on peach. Based on the *G3PDH* gene sequences, primer pair PRCmon-F/PRCmon-R amplified a 354 bp fragment from all *Monilinia* species, but not from closely related genera such as *Botrytinia* and *Sclerotinia* (Fig. 8). The cocktail also contained common reverse primer Mon-R and forward species-specific primers *Cola*-F, *Ensis*-F, and *Mume*-F, which produced amplicons 237 bp, 534 bp, or 712 bp in length from *M. yunnanensis*, *M. fructicola*, or *M. mumecola*, respectively (Fig. 8). All previously confirmed *Monilinia* isolates used in this study (Table S1) produced the expected amplicon sizes with this new multiplex PCR tool. All 145 isolates collected for this study from China were identified as *M. fructicola*, *M. yunnanensis*, or *M. mumecola*. Isolates that were collected from Beijing, Shandong, Zhejiang, Fujian and Gansu provinces were *M. fructicola*, isolates from Hubei province in central China were *M. mumecola*, and isolates from Yunnan and Shanxi provinces in Western China were *M. yunnanensis*. The closely related *B. fuckeliana* and *S. sclerotiorum* were also tested using these two primer sets. While there was no amplicon obtained from *S. sclerotiorum*, a 237 bp fragment was produced from *B. fuckeliana*. Although, this 237 bp amplicon was also produced in *M. fructicola*, *B. fuckeliana* produces no amplicon with the PRCmon-F1/PRCmon-R1 primer set (Fig. 8).

## Discussion

Our phylogenetic, morphological, and cultural characterization of *Monilinia* isolates suggested that in our sample size there were three species causing brown rot of peach and nectarine in China, one of which we believe to be a new species *M. yunnanensis*. The ITS sequence is widely used in taxonomy and molecular phylogeny [34], [35], [36], and several phylogenetic analyses had been performed among and within *Monilinia* species on the basis of sequence differences in this region [33], [37], [38]. However, we found this locus to provide a fairly weak phylogeny of *Monilinia* species evidenced by the low bootstrap values resulting from the relative low number of parsimony informative positions. Hence, we constructed addiltional phylogenies from *TUB2*, and *G3PDH*, which have also revealed significant taxonomic relations in fungi [39], [40], [41], [42], [43], [44]. For examples, Couch and Kohn [39] segregated *Magnaporthe oryzae* from *M. grisea* using a multilocus gene genealogy, including the *TUB2* sequences; Fournier et al. [45] distinguished two *Botrytis* sibling species (*B. cinerea* Group I and II) from grape by using DNA sequence data including the *TUB2* and *G3PDH* genes. The ITS, *TUB2*, and *G3PDH* data in our study clearly support the designation of a new *Monilinia* species, *M. yunnanensis*. Morphological data as well as in vitro growth characteristics and pathogenicity data are concordant with the molecular evidence for the suggested species delineation.

ITS sequence data showed that *M. yunnanensis* was distantly related to *M. polystroma*, a species shown to affect *Malus*, *Pyrus*, and *Prunus* spp. (but not *P. persica*) and which was found in China only recently [7]. *M. polystroma*, which is most closely related to *M. fructigena*, was designated as a new species based on its ability to produce more stroma and based on five basepair differences in the ITS regions compared to *M. fructigena* [12]. In comparison, *M. yunnanensis* revealed eleven nucleotide variations in the ITS compared to its respective closest *Monilinia* relative *M. fructigena*.

In addition to the phylogenetic support, morphological data also distinguished *M. yunnanensis* from the closely related *M. fructigena*. For example, the colony morphology was unlike that of *M. fructigena*, and *M. yunnanensis* conidia were smaller than those of *M. fructigena*. Previous studies [37], [46], [47], [48] have acknowledged that what was believed to be populations of *M. fructigena* from Asia were different from European *M. fructigena* populations. Indeed, Khokhriakova [46] found differences in conidia size between *M. fructigena* populations from Europe and different regional populations in the former Soviet Union. The author found that the mean length and width of conidia were 19.4×11.5 µm for isolates from a European population, 17.2×11.9 µm for isolates from a central Asian population, and 18×11.4 µm for isolates from a Far Eastern population. Leeuwen [12] also made similar observations and found that conidia from Asian isolates tended to be smaller. Wormald [47] noted that a Japanese *M. fructigena* culture produced zones of black stromatal plates in culture, but the author never observed this phenomenon in European *M. fructigena* isolates. Willetts [48] found that European *M. fructigena* isolates produced few poorly-developed stromatic tissue, compared with Asian *M. fructigena* isolates. Consistent with this study, considerable numbers of stromata were produced in most *M. yunnanensis* isolates. Given the considerable evidence, it is likely that what was believed to be *M. fructigena* from Asia was likely a new species, but not realized.

The already strong phylogenetic and morphological evidence outlined in this study for delineating *M. yunnanensis* from *M. fructigena* was further supported by analysis of the cytochrome b gene sequence. The *Cyt b* gene exon/intron organization of *M. yunnanensis* was quite different from *M. fructigena*. Only four of the seven introns in *M. yunnanensis* revealed sequence identities greater than 98% compared to the corresponding introns in *M. fructigena*. The coding region of the *Cyt b* gene from *M. yunnanensis* showed 99.1% identity with that of *M. fructigena*. This high level of homology is to be expected given that the *Cyt b* exon sequences in general were highly conserved among the five *Monilinia* species for which the gene has been cloned [32]. For example, the authors found that *M. fructicola* and *M. fructigena* exhibited 97.5% sequence identity the least, while *M. laxa* and *M. fructigena* displayed more than 99.1% sequence identity.

Based on both molecular and morphological evidence we propose to name *M. yunnanensis* its own species. Given that only the anamorph has been observed, we describe the anamorph of *Monilia yunnanensis*.

### *Monilia yunnanensis* M.J. Hu & C.X. Luo, sp. nov

MycoBank no.: MB563122.

Etym. “*yunnanensis*” indicates the province (Yunnan), where the fungus was originally isolated.

Colonies on potato dextrose agar (PDA) and on V8 reaching 50–70 mm, 75–80 mm in diameter respectively after 7 d at 22°C. When grown on PDA, colonies begin pale green and become tan after 15–20 days of incubation at 22°C, conidia sparse, stromata abundant, black in color, spherical to elliptical in shape, discrete or aggregated. Conidia ovoid or limoniform, measuring 10–21×7–12 µm, av. 15×9 µm when grown on peach fruit at 22°C. Mycelial pustules are common on symptomatic fruits.

Living culture YKG10-61c (AF2011002, CCTCC) was deposited in the China Center for Type Culture Collection (CCTCC) at Wuhan University, Wuhan City, Hubei Province, China. This single-spore isolate was collected from a peach fruit in Anning City, Yunnan Province, China, on August 5, 2010.

Despite attempts (not shown), we were not able to induce apothecium production in *M. yunnanensis* following the method described previously [49]. Mummified peach fruit infected by *M. yunnanensis* were collected to induce the production of apothecia using the method for *Monilinia vaccinii-corymbosi* [50]. However, no apothecia were produced from fruit infected with *M. yunnanensis* using this method. Given that no teleomorph examples are currently available, we only describe a new anamorphic species.

In regard to *M. mumecola*, one of the other species in our survey, the phylogenetic and morphological data (ITS1-5.8S-ITS2 sequences, conidium size, germ tube development, and colony appearance on PDA) were consistent with the previous description of the species [15]. However, to our knowledge this is the first report of *M. mumecola* causing brown rot of peach and nectarine in China.

*M. fructicola* was the third species we routinely identified in our survey. This fungus was only recently reported a new pathogen in China [6], [11], however, a population analysis indicated that this species had been in the country long before the first report in 2005 [51]. In our study, *M. fructicola* was collected from most provinces investigated, which supports the conclusions made by Fan et al. (2010) that this species has been in China for a considerable period of time. In both of our phylogenetic analyses, the *M. fructicola* isolates from China and America clustered together and this close relationship was further supported by morphological and cultural characteristics. This species was most distantly related to *M. laxa* and *M. mumecola*.

In contrast to other studies [8], [52], we did not find evidence for the presence of *M. fructigena* and *M. laxa* on peach or nectarine in China. Although isolates were collected from nearly all major peach growing provinces including Northern China (Beijing), Eastern China (Shandong), Southeastern China (Zhejing, Fujian), Central China (Hubei), Northwestern China (Shanxi, Gansu), and Southwestern China (Yunnan), our sample size of 145 isolates may not be sufficient to rule out the existence of *M. fructigena* and *M. laxa* in China.

We developed a new PCR-based method for differentiating *Monilinia* spp. infecting peach in China. Aforementioned studies [8] used three molecular techniques to identify Chinese isolates of *Monilinia* to the species level. We've demonstrated that techniques, developed primarily to distinguish European and North American *Monilinia* species, do not accurately discriminate *Monilinia* species from China, especially *M. yunnanensis*. Moreover, using previously developed techniques, we've found that *M. yunnanensis* and *M. mumecola* can be misidentified as *M. fructigena* and *M. laxa*, respectively. For example, our phylogenetic analysis of the ITS, *G3PDH* and *TUB2* sequences confirmed that *M. mumecola* is closely related to *M. laxa*, which supports of the possibility of prior misidentification. Additionally, the cultural characteristics on PDA medium, including growth rate, colony morphology, and sporulation, largely matched those described for *M. laxa* by Leeuwen and Leeuwen [53] and Lane [54].

The phylogenetic analysis of ITS, *G3PDH* and *TUB2* gene sequences allows some speculation on the evolution of the *Monilinia* species. *M. mumecola* appears to be a direct descendant of *M. laxa*. Both phylogenetic trees also indicate a close relationship between *M. yunnanensis* and *M. fructigena* suggesting that *M. fructigena* may have evolved from *M. yunnanensis*. In contrast to a previous rDNA analysis [33], our phylogenetic analyses of *G3PDH* plus *TUB2* gene sequences illustrated that *M. fructicola* may have evolved earlier than other *Monilinia* spp. The latter hypothesis is strengthened by the fact that the rDNA sequences contained less phylogenetically informative characters compared with the *G3PDH* and *TUB2* sequences. A more detailed DNA sequence analysis, including additional populations, populations from other parts of world, and additional genes, would provide a more complete picture of the evolution of *Monilinia* species. Leeuwen [12] suggested that the ancestor of *M. fructigena* and *M. polystroma* might have evolved in the Far East as a specialized fruit pathogen, before they evolved into two distinct groups in Europe and Japan due to geographical separation. The fact that *M. yunnanensis* found in China appears to be more basal in the phylogeny than *M. fructigena*, lends some support to Leeuwen's suggestion.

In conclusion, phylogenetic, morphological, and cultural analysis of *Monilinia* isolates from China revealed a previously undescribed species we've designated *Monilia yunnanensis*, and the first report of *M. mumecola* on peach and nectarine. A molecular assay was developed to detect these two species and differentiate the Chinese *Monilinia* species pathogenic on peach including the newly described *M. yunnanensis*.

## Supporting Information

Table S1 **Isolates utilized in this study.**
(DOC)
