# Supplementary material for: Monilinia Species Causing Brown Rot of Peach in China
Source: PLoS One. 2011 Sep 27;6(9):e24990. doi: 10.1371/journal.pone.0024990 (PMC3181254; doi:10.1371/journal.pone.0024990)
Supplement: Table S1 — Isolates utilized in this study. (DOC) [file pone.0024990.s001.doc]

**Supporting information:**

**Table S1**. Isolates utilized in this study

| Taxon | Isolate name^z^ | Origin | | Host | Isolation year |
| --- | --- | --- | --- | --- | --- |
|  |  | Country | City, state |  |  |
| *Monilinia fructicola* |  |  |  |  |  |
|  | MSA9 | China | Shunyi, BJ | Peach | 2007 |
|  | MSB10* | China | Shunyi, BJ | Peach | 2007 |
|  | MBJA8 | China | Pinggu, BJ | Peach | 2007 |
|  | 0907-a* | China | Fuzhou, FJ | Peach | 2009 |
|  | 0908-b | China | Fuzhou, FJ | Peach | 2009 |
|  | ZM09-2a* | China | Hangzhou, ZJ | Peach | 2009 |
|  | ZM09-4a | China | Hangzhou, ZJ | Peach | 2009 |
|  | YM09-1b* | China | Kunming, YN | Peach | 2009 |
|  | SD-5a* | China | Weifang, SD | Peach | 2009 |
|  | MF-2 | China | Wuhan, HB | Peach | 2009 |
|  | GA.Bmpc5* | USA | Byron, GA | Peach | 2006 |
|  | SC.Egpc8* | USA | Edgefield, SC | Peach | 2007 |
|  | SC.Dap3* | USA | Spartanburg, SC | Peach | 2008 |
| *Monilia yunnanensis* |  |  |  |  |  |
|  | QJ-2a* | China | Qujing, YN | Peach | 2009 |
|  | QJ-4a* | China | Qujing, YN | Peach | 2009 |
|  | KY-1* | China | Kaiyuan, YN | Peach | 2009 |
|  | SM09-1a | China | Baoji, SX | Peach | 2009 |
|  | SM09-5a* | China | Baoji, SX | Peach | 2009 |
|  | SM09-7a* | China | Baoji, SX | Peach | 2009 |
|  | SBG10-3a* | China | Baoji, SX | Peach | 2010 |
|  | YQG10-6c* | China | Qujing, YN | Peach | 2010 |
|  | YKG10-61c* | China | Kunming, YN | Peach | 2010 |
| *Monilinia fructigena* |  |  |  |  |  |
|  | ES49* | Spain | N/A | Plum | 1996 |
|  | Apple 15* | England | N/A | Apple | 1999 |
|  | Mfg2-GE-A* | Hungary | N/A | Apple | 2009 |
|  | Mfg4-GY-A* | Hungary | N/A | Apple | 2009 |
|  | Mfg5-SP-A | Hungary | N/A | Apple | 2009 |
|  | SL10* | Germany | N/A | Peach | 2010 |
| *Monilia mumecola* |  |  |  |  |  |
|  | ML-1a* | China | Wuhan, HB | Peach | 2008 |
|  | ML-1c* | China | Wuhan, HB | Peach | 2008 |
|  | HWL10-1b* | China | Wuhan, HB | Peach | 2010 |
|  | HWL10-13b* | China | Wuhan, HB | Peach | 2010 |
|  | HGL10-1a* | China | Xiaogan, HB | Peach | 2010 |
|  | HXL10-1a* | China | Xiangfan, HB | Nectarine | 2010 |
|  | HXL10-4a* | China | Xiangfan, HB | Nectarine | 2010 |
| *Monilinia laxa* |  |  |  |  |  |
|  | EBR Ba11b* | USA | Niagara, NY | Cherry | 2008 |
|  | EBR A-W-1* | USA | Niagara, NY | Cherry | N/A |
|  | B. Sch cal* | USA | Niagara, NY | Cherry | 2009 |
|  | GEARI 3c2a* | USA | Middleton, RI | Cherry | N/A |
|  | GEARI 6a3c* | USA | Middleton, RI | Cherry | N/A |
|  | L14* | Spain | N/A | N/A | N/A |
|  | BSZGY-SZ-1* | Hungary | Győröcske | Plum | 2009 |
|  | BEK-SZ* | Hungary | Eperjeske | Plum | 2009 |
|  | BF-SZ-1* | Hungary | Földes | Plum | 2009 |

^z^ symbol ‘*’ indicates isolate was used for phylogenetic analysis.
